# Identification and molecular characterization of a novel *Babesia orientalis* thrombospondin-related anonymous protein (BoTRAP1)

**DOI:** 10.1186/s13071-018-3245-2

**Published:** 2018-12-27

**Authors:** Long Yu, Qin Liu, Xueyan Zhan, Yuan Huang, Yali Sun, Zheng Nie, Yangnan Zhao, Xiaomeng An, Muxiao Li, Sen Wang, Yangsiqi Ao, Cuiqin Huang, Lan He, Junlong Zhao

**Affiliations:** 1 State Key Laboratory of Agricultural Microbiology, College of Veterinary Medicine, Huazhong Agricultural University, Wuhan, 430070 Hubei China; 2 Key Laboratory of Preventive Veterinary Medicine in Hubei Province, Wuhan, 430070 Hubei China; 3 College of Life Science, Longyan University, Longyan, 364012 Fujian China; 4 Fujian, Provincial Key Laboratory for the Prevention and Control of Animal Infectious Diseases and Biotechnology, Longyan, 364012 Fujian China; 5 Key Laboratory of Animal Epidemical Disease and Infectious Zoonoses, Ministry of Agriculture, Huazhong Agricultural University, Wuhan, 430070 Hubei China

**Keywords:** *Babesia orientalis*, Thrombospondin-related anonymous protein 1, Microneme protein, Babesiosis

## Abstract

**Background:**

The thrombospondin-related anonymous protein (TRAP) family, a kind of transmembrane protein, is widely distributed with a conserved feature of structure in all apicomplexan parasites and plays a crucial role in the gliding motility and survival of parasites.

**Methods:**

The *Babesia orientalis TRAP1* gene (*BoTRAP1*) was truncated and cloned into a pET-42b expression vector and expressed as a GST-tag fusion protein with a TEV protease site. Rabbit anti-rBoTRAP1 antibody was produced and purified using a protein A chromatography column. Western blot analysis was performed to identify the native protein of BoTRAP1 and differentiate *B. orientalis*-infected positive from negative serum samples. The localization of BoTRAP1 on merozoites was identified by the indirect florescent antibody test (IFAT).

**Results:**

The partial sequence of the *TRAP1* gene was cloned from *B. orientalis* cDNA and identified to contain a von Willebrand factor A (vWFA) region and a thrombospondin type-1 (TSP-1) domain; it had a length of 762 bp, encoding a polypeptide of 254 amino acid residues with a predicted size of 28.2 kDa. The partial sequence was cloned into a pET-42b expression vector and expressed in *E. coli* as a GST fusion protein. Western blot indicated that rBoTRAP1 has a high immunogenicity and can differentiate *B. orientalis*-infected positive and negative serum samples collected from water buffaloes. IFAT showed that BoTRAP1 is mainly localized on the apical end of intracellular parasites by using polyclonal antibodies (PcAb) against rBoTRAP1. Meanwhile, the PcAb test also identified the native BoTRAP1 as a ~65 kDa band from *B. orientalis* lysates. The predicted 3D structure of BoTRAP1 contains a metalion-dependent adhesion site (MIDAS), which could be important for interaction with ligand on the surface of the host cells.

**Conclusions:**

Like all known protozoa, *B. orientalis* has a TRAP family, comprising TRAP1, TRAP2, TRAP3 and TRAP4. The newly identified and characterized BoTRAP1 may play a key role in the invasion of *B. orientalis* into water buffalo erythrocytes.

## Introduction

*Babesia orientalis* is an apicomplexan parasite that is widespread in southern China and causes babesiosis in water buffaloes, leading to an enormous economic loss [1, 2]. The clinical symptoms in water buffalo include anemia, fever, icterus, hemoglobinuria and even death [2, 3]. Currently, no vaccine is available to control *B. orientalis* infection, and drugs for treating *B. orientalis* are also scarce, suggesting the importance and necessity to explore potential vaccines based on related antigen molecules.

All the thrombospondin-related anonymous protein (TRAP) family members are secreted by micronemes as a membrane protein, and TRAPs with conserved structures are present in all protozoans, with one or more von Willebrand factor A (vWFA) and thrombospondin type-1 repeat (TSR) domain in their extracellular region, as well as a cytoplasmic tail domain (CTD) with a tryptophan residue [4]. In malaria parasites, the TRAPs were first identified in *Plasmodium falciparum*, and their homologues were found in all other *Plasmodium* species [5, 6]. Subsequent studies have shown that the TRAPs are expressed in different plasmodial stages, such as sporozoite, merozoite and ookinete, and their orthologues are also present in other protozoa, including *Toxoplasma gondii*, *Neospora caninum*, *Babesia* spp., *Cryptosporidium* spp. and *Eimeria* spp. [7, 8]. In *B. bovis* and *B. gibsoni*, the TRAPs were only expressed during the asexual stage, and the antibodies of their recombinant proteins have an obvious influence on *Babesia* invasion into the host red blood cells (RBCs) [9, 10].

In the life-cycle of apicomplexan parasites, host cell invasion is a crucial step for survival, and the process is highly dependent on the interaction between the parasite- and host-surface molecules [11]. In *Plasmodium* spp., the first step in the invasion of the extracellular merozoites is the attachment to the host cells. In this process, the initial adhesion with host cells based on glycosyl phosphatidylinositol anchor (GPI) of merozoite surface proteins (MSPs) is invertible, followed by re-orientation to link the anterior tip of merozoites with the plasma membrane of host cells, leading to the formation of tight junctions from higher-affinity transmembrane proteins secreted by micronemes and rhoptries of parasites; this attachment to the surface of host cells is irreversible. Finally, the parasites invade host cells *via* a moving complex that involves both apical membrane antigen 1 (AMA1) and rhoptry neck proteins (RONs); this motor process is driven by an actomyosin motor [12]. During the invasion, TRAPs play an important role in the formation of actomyosin motor by linking to actin through their cytoplasmic tail domains (CTD) while binding to host cells *via* their vWFA domains [7, 13]. Subsequent studies have demonstrated that the interaction between TRAP CTD and actin-myosin is connected by aldolase and depends on the sub-terminal tryptophan residue of cytoplasmic tail [14].

Currently, vaccine development efforts have shifted toward the use of antigenically defined immunogens, particularly the molecules interacting or disrupting the process of parasite invasion into host RBCs [10, 15–17]. Therefore, identification and characterization of these genes encoding TRAPs in *Babesia* spp. would facilitate the discovery of novel *Babesia* vaccine candidate antigens.

## Methods

### Parasites

*Babesia orientalis* (Wuhan strain) was isolated from Wuhan city, Hubei Province, China, and preserved in liquid nitrogen with the additive of dimethyl sulfoxide (DMSO) in the State Key Laboratory of Agricultural Microbiology, Huazhong Agricultural University, China.

Two water buffaloes (1.5 years-old) were purchased from a *Babesia*-free area, and were confirmed to be free of *Babesia* and *Theileria* by microscope examination and real-time PCR [18]. The water buffalos were splenectomized two weeks before injection of 4 ml of *B. orientalis* infected blood with the percentage of parasitized erythrocytes (PPE) being 1%. Blood samples were collected every day to monitor the parasitemia until reaching 3%.

### Preparation of RNA and cDNA

Blood from the jugular vein of experimentally infected water buffaloes was collected in 50 ml centrifuge tubes containing EDTA-K_2_ (Sigma, Shanghai, China). Total RNA was extracted from purified *B. orientalis* merozoites by using the TransZol Up (TransGen Biotech, Beijing, China) and treated with RNase-free DNaseI (TAKARA, Dalian, China). The cDNA was prepared from 1 μg of the total RNA using PrimeScript^TM^ RT reagent Kit with gDNA eraser (TAKARA, Dalian, China) according to the manufacturer’s instructions.

### Preparation of recombinant plasmid

Primer pairs for the full-length and partial *BoTRAP1* sequences including a vWFA region and a TSP-1 domain were designed based on the fragment of *BoTRAP1* screened from *B. orientalis* genome database (Table 1). The PCR reaction was performed using the following cycling parameters: 94 °C for 5 min, followed by 35 cycles (94 °C for 30 s, 58 °C for 30 s, 68 °C for 1 min), and a final extension at 68 °C for 10 min. The resulting PCR product was purified by using EasyPure® PCR Purification Kit (TransGEN, Beijing, China), and then cloned into a pET-42b expression vector. All the constructs were confirmed by DNA sequencing.

**Table 1:** Oligonucleotide primers used for the amplification of the full-length and partial BoTRAP1 genes

Primer	Sequence (5'-3')	Restriction enzyme
TRAP1-F (full)	ATGATTGGTTACAACAAAATTTGGGGCTACG	
TRAP1-R (full)	TTAGGCTGCTTCACCCCAAATGTTATTGTC	
TRAP1-F (partial)	TCACTAGTGAAAACCTGTATTTTCAGGGC (TEV protease site) CTCGACTTCTCCATCGTGG	*Spe*I
TRAP1-R (partial)	CGCTCGAGTTAGTGGCATTTTTTAATACACCCCTCTGAC	*Xho*I

### Protein expression and purification

The recombinant plasmid was transformed into *E. coli* BL21 expression host cells, followed by incubation at 37 °C in LB medium containing 100 mg/ml kanamycin for 3 h. At an optical density of 0.6 to 0.8 at 600 nm (OD_600_), the cells were induced with 0.8 mM IPTG (Biosharp, Anhui, China), further cultured at 25 °C for another 12 h and then harvested.

For protein purification, cells were harvested by centrifugation at 7000 rpm for 10 min in a high-speed refrigerated centrifuge (Hitachi, Tokyo, Japan), resuspended in PBS (pH 7.5) and lysed by passing through a high-pressure homogenizer at 1000 Bar. After centrifugation at 10,000× *rpm* and 4 °C for 10 min, the supernatant was filtered through a 0.45 μm pore size filter and loaded onto glutathione sepharose beads (GE Healthcare, Uppsala, Sweden). The proteins were eluted with elution buffer (9.5 ml ddH_2_O, 50 mM Tris-HCl, 10 mM L-glutathione reduced glutathione). The GST-tag on the N-terminus was cleaved using TEV (Tobacco Etch Virus) protease (Solarbio, Shanghai, China) at 4 °C for 8 h.

### Preparation of anti-rBoTRAP1 immune serum

Polyclonal antibodies against BoTRAP1 protein were prepared in Japanese white rabbits (*n* = 2) according to the established immune procedure. Briefly, seven days after purchase, the rabbits were subcutaneously immunized with the purified 500 μg recombinant protein emulsified in equal amounts of Freund’s complete adjuvant (Sigma, Shanghai, China), followed by a second immunization with one half of the same recombinant protein emulsified in equal amounts of Freund’s incomplete adjuvant (Sigma, Shanghai, China) at a 14-day interval. This was followed by another three immunizations each at 7-day intervals. After the titer of the antisera was assayed by ELISA (the recombinant protein was coated), blood samples were collected from the carotid artery of the rabbits to prepare the polyclonal antibodies. Total immunoglobulin Gs (IgGs) were purified from rabbit sera through a Protein A chromatography column according to the manufacturer’s instructions (Beyotime Biotechnology, Shanghai, China).

### Identification of immunogenicity and native BoTRAP1 by Western blot

The *B. orientalis-*infected RBCs (1 ml) were suspended in an equal amount of phosphate-buffered saline (PBS) buffer, and supplemented with red blood cell lysis buffer preheated at 37 °C (18 ml), followed by heating in a water bath for 5 min and centrifugation at 2450× *rpm* for 5 min. The resulting supernatant was collected and centrifuged at 12,000× *rpm* for 20 min. After a high-speed centrifugation, pellets (parasite fraction) from previous steps were collected and washed three times in cold phosphate-buffered saline (PBS) buffer, followed by the addition of an equal amount of PBS and centrifugation at 15,000× *rpm* for 20 min. After discarding the supernatant, the pellets were suspended in PBS and stored at -20 °C.

The rBoTRAP1 and *B. orientalis* lysates were separately subjected to 12% SDS-PAGE using the standard method, followed by electroblotting onto a nitrocellulose membrane. The membranes for the Western blot were blocked with 0.05% Tween-20 in TBS (TBST) plus 1% BSA overnight at 4 °C, and then separately probed with the anti-BoTRAP1 PcAb or positive serum of *B. orientalis* diluted with TBST (1:200) at 37 °C for 1 h. The membranes were washed 5 times in TBST and incubated with secondary antibodies diluted with TBST (1:1000, HRP labeled goat anti-rabbit IgG) at 37 °C for 1 h. After rinsing 5 times in TBST, the positive bands on membranes were visualized using the ECL method.

### Localization of BoTRAP1 by indirect florescent antibody test (IFAT)

The *B. orientalis*-infected water buffalo RBCs (3% parasitemia) were smeared on glass slides and fixed in cold 100% methanol (-20 °C). After three washes, blood smears were permeabilized by using 0.1% Triton-100 for 30 min, followed by incubation separately with anti-BoTRAP1 PcAb and pre-immune rabbit sera diluted 200× with 1× PBS/1% BSA for 30 min. The secondary antibody was anti-rabbit Alexa-594 diluted 1000× with 1× PBS/1% BSA and the parasite nucleus was stained with a Hoechst stain. Finally, the coverslips with cells were mounted on a slide in 10 μl anti-fluorescence quenching agent and observed with a confocal laser scanning microscope.

### Sequence analysis and crystal structure modeling of BoTRAP1

The *BoTRAP1* gene has a significant degree of similarity to the previously reported *BbTRAP1* (XM_001609738.1), and was analyzed by Conserved Domain Search Service (CD Search) of NCBI (https://www.ncbi.nlm.nih.gov/Structure/cdd/wrpsb.cgi) for the presence of vWFA and TSP-1 domains. Sequence alignment and phylogenetic analysis of *BoTRAP1* with related apicomplexan parasites was performed using the MEGA7 software. The BoTRAP1 3D structure model was generated using SWISS-MODEL (https://swissmodel.expasy.org/interactive), according to the reported crystal structure of *Plasmodium vivax* TRAP protein (PDB code: 4hqo).

## Results

### Characterization of *BoTRAP1* gene

**Fig. 1:**
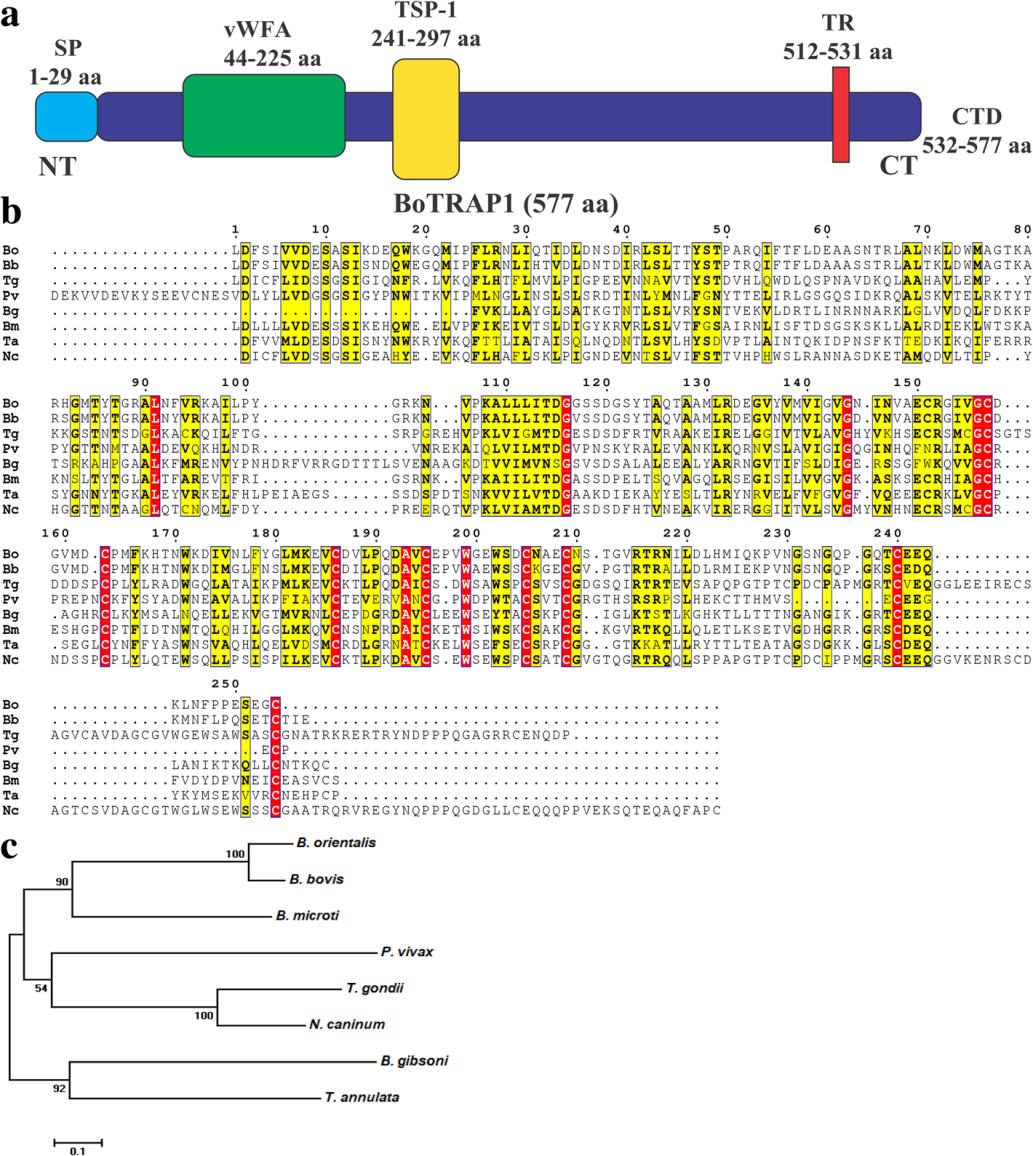
Alignment of amino acid sequences and phylogenetic analysis of TRAPs. **a** Graphic depiction of BoTRAP1. A signal peptide at the N-terminus, two functional domains (vWFA and TSP-1), a transmembrane region and a cytoplasmic C-terminal tail domain were dispersed in the coding region. **b** *BoTRAP1* sequence alignment across various strains of apicomplexan parasites, and the sequences were aligned using ClustalW. **c** Neighbor-joining tree showing phylogenetic relationship of the BoTRAP1 sequences of two conserved regions identified in this study, with sequences of other apicomplexan parasite. The scale-bar represents the nucleotide substitutions per position. Branch lengths represent the amount of genetic distance change between the strains. *Abbreviations*: SP, signal peptide; TR, transmembrane region; CTD, C-terminal tail domain

The cDNA sequence of *BoTRAP1* had a length 1734 bp, contained five exons and encoded a polypeptide of 577 amino acid residues with a signal peptide and transmembrane region (Fig. 1a). Multiple sequence alignment of the two *BoTRAP1* domains with the related *TRAP* domains of apicomplexan parasites revealed that the two domains from *BoTRAP1* have a low similarity, 29%, 28%, 22%, 40%, 30% and 30%, to those of *Toxoplasma gondii* (GenBank: AAB63303.1), *Plasmodium vivax* (GenBank: AAC97484), *Babesia gibsoni* (GenBank: BAI66064.1), *Babesia microti* (GenBank: XP_012650313.1), *Theileria annulata* (GenBank: XP_952976.1) and *Neospora caninum* (GenBank: AAF01565.1), respectively. However, they have a high similarity of 80% with the two domains of *Babesia bovis* (GenBank: XP_001609788.1) (Fig. 1b). Phylogenetic analysis based on vWFA and TSP-1 showed that *B. orientalis* has a closer relationship with *B. bovis* than the other apicomplexan parasites (Fig. 1c).

### Expression of the recombinant BoTRAPl

**Fig. 2:**
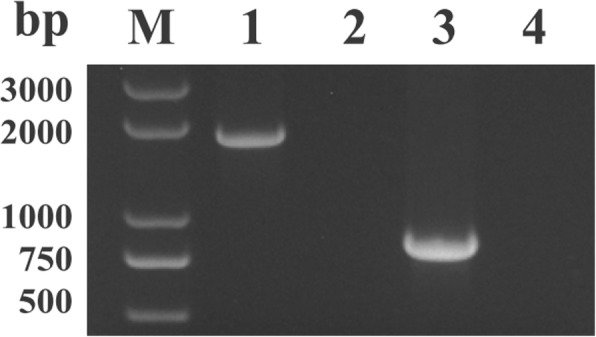
PCR results of amplifying the full-length and the partial sequence of *BoTRAP1* from *B. orientalis* cDNA. Lane M: molecular weight marker; Lane 1: the full-length *BoTRAP1* (1734 bp); Lane 2: negative control; Lane 3: the partial *BoTRAP1* including vWFA and TSP-1 domains (762 bp); Lane 4: negative control

**Fig. 3:**
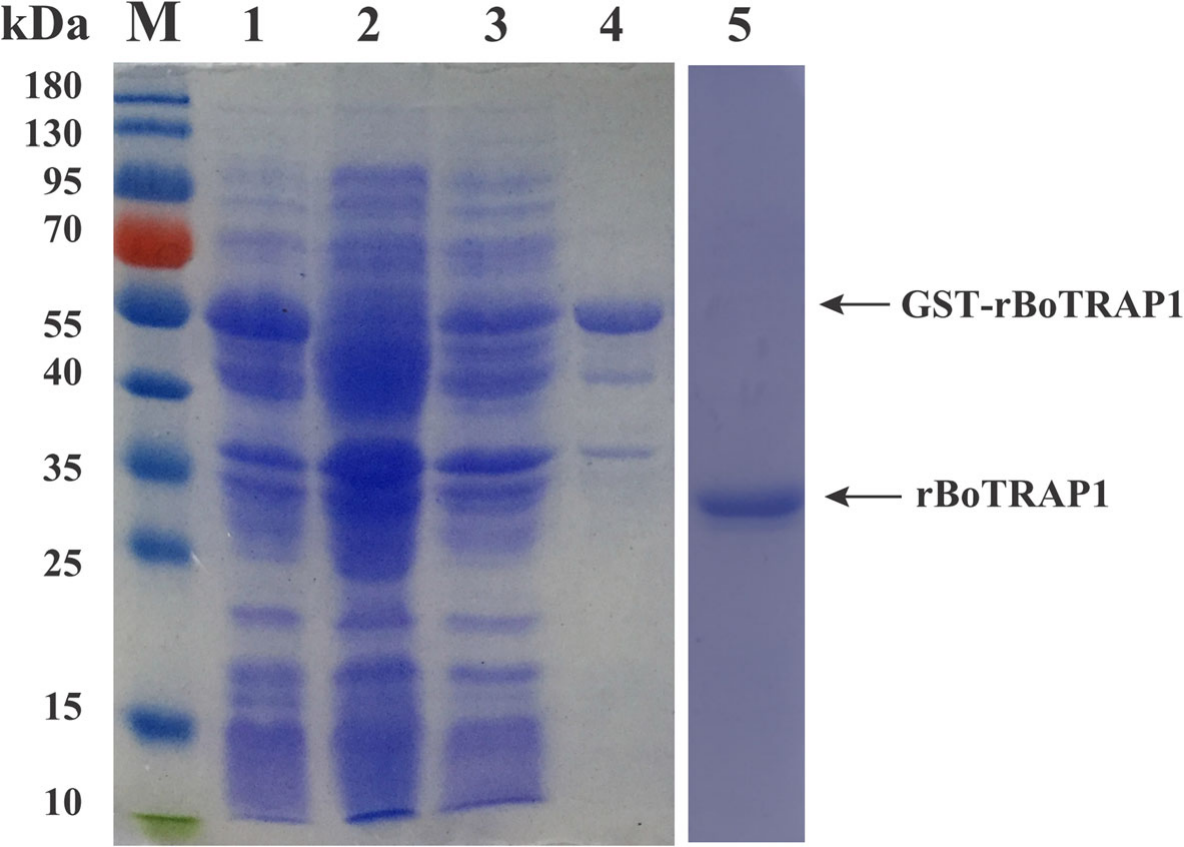
SDS-PAGE analysis of bacterial recombinant stained by Coomassie blue. Lane 1: induced protein; Lane 2: non-induced control; Lane 3: soluble GST-BoTRAP1 in cell lysates; Lane 4: purified GST-BoTRAP1; Lane 5: rBoTRAP1 cleaved by using TEV protease

The full and partial *BoTRAP1* sequences were obtained separately from *B. orientalis* cDNA by PCR (Fig. 2). For molecular characterization of BoTRAP1, the truncated fragment was expressed in *E. coli* with a TEV protease site, and the size of rBoTRAP1 was ~55 kDa including a 26 kDa GST-tag (Fig. 3, Lane 1). The rBoTRAP1 was purified by glutathione sepharose beads (Fig. 3, Lane 4), and the GST tag on the N-terminus was removed by using the TEV protease (Fig. 3, Lane 5).

### Immune reactivity of rBoTRAP1 and identification of native BoTRAP1

**Fig. 4:**
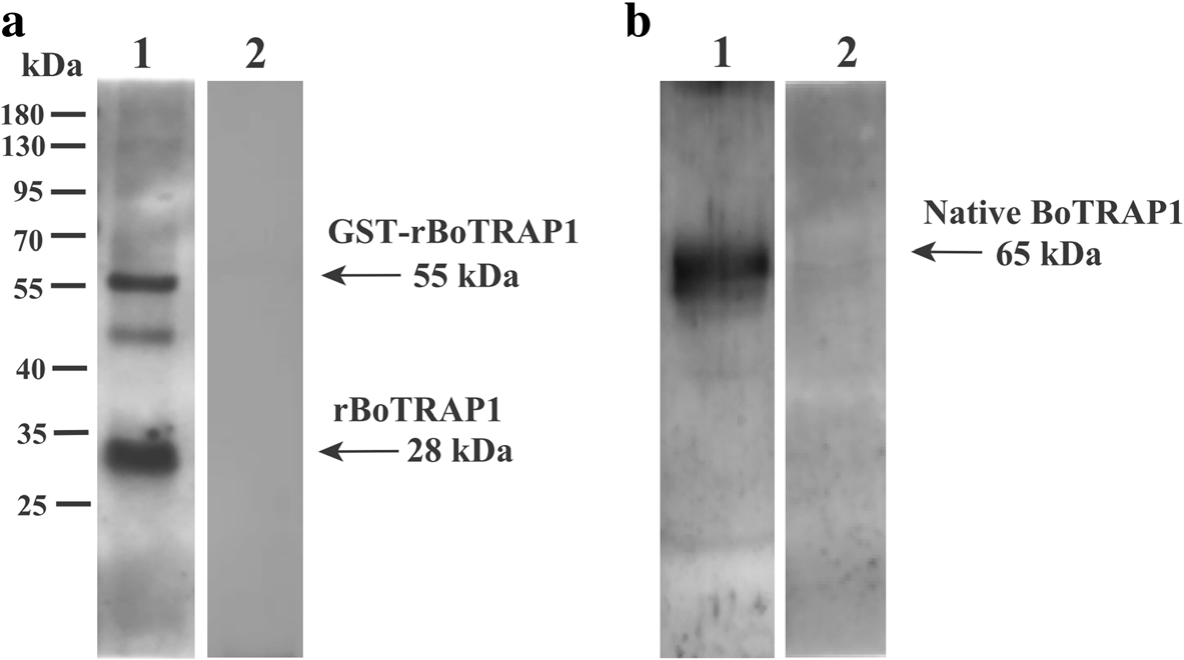
Western blot analysis of BoTRAP1. **a** Determination of antibody response of rBoTRAP1 in water buffalo antiserum. Lane M: molecular weight marker; Lane 1: rBoTRAP1 reacted with *B. orientalis* positive serum; Lane 2: rBoTRAP1 probed with *B*. *orentalis* non-infected serum from water buffalo. **b** Determination of native BoTRAP1 in the merozoite stage. *B. orientalis* lysates (Lane 1) and non-infected bovine RBCs lysates (Lane 2) were probed with anti-BoTRAP1 rabbit serum

**Fig. 5:**
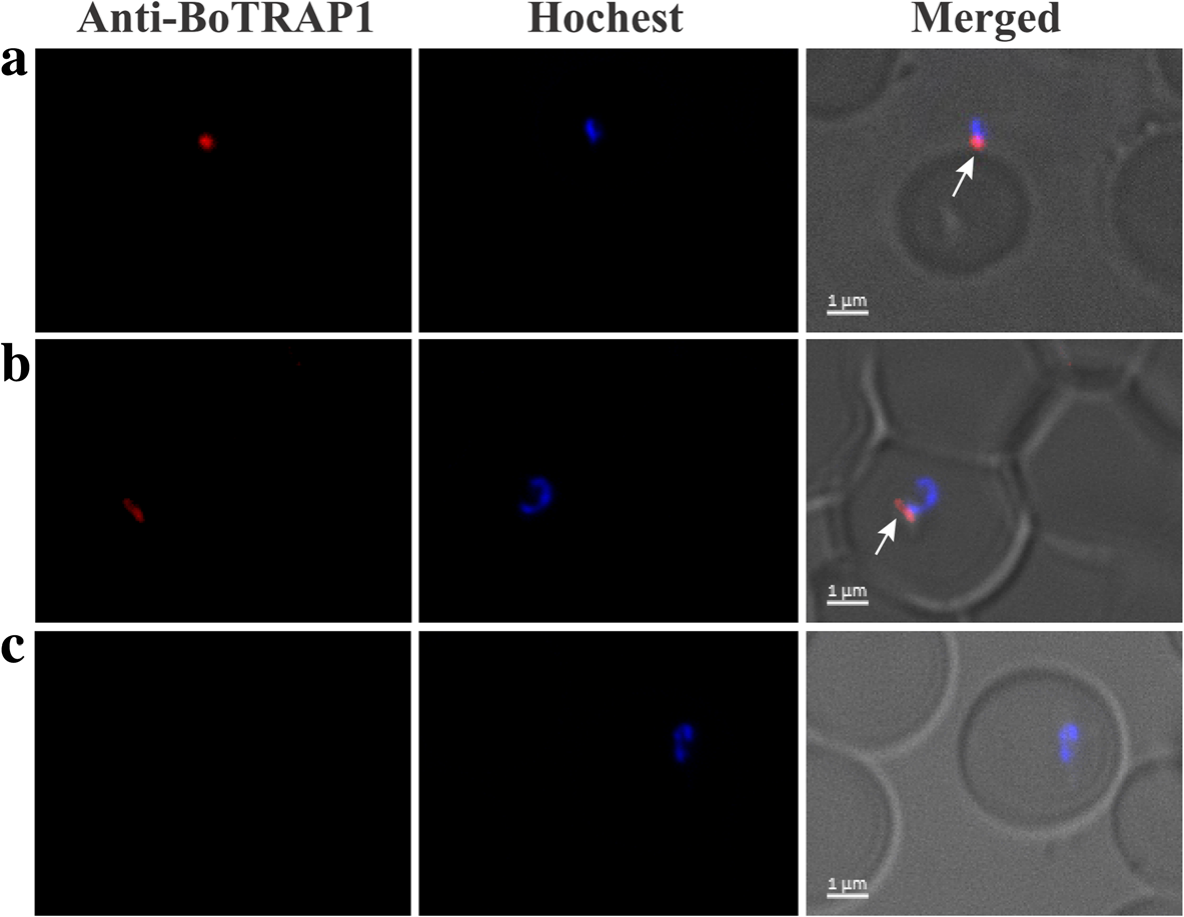
Localization of BoTRAP1 on *B. orientalis* by immunofluorescence analysis. *Babesia orentalis-*infected RBCs were stained separately with PcAb-BoTRAP1 and pre-immune serum. Reactivity of the anti-rBoTRAP1 serum with extracellular parasite (**a**) and intracellular parasite single (**b**). Pre-immune sera were used as negative control for the validation test (**c**). PcAb-BoTRAP1 (Red) reacted with native TRAP1 on merozoites. Nuclei were counterstained by Hochest (blue). *Scale-bars*: 1 μm

The potential of BoTRAP1 as vaccine candidate antigen for preventing and controlling *B. orientalis* infection in water buffalo was tested by Western blot, and two bands corresponding to 55 kDa (GST fusion) and 28 kDa (GST removed) rBoTRAP1 were detected by using positive serum in contrast to no signal observed with negative sera used as control (Fig. 4a). The results indicated that rBoTRAP1 could differentiate *B. orientalis* positive and negative serum samples from water buffalo. To identify native BoTRAP1 in *B. orientalis*, anti-rBoTRAP1 immune sera were produced in rabbits, purified with a Protein A chromatography column, and used to detect *B. orientalis* lysates by Western blot. One band corresponding to ~65 kDa native BoTRAP1 was detected (Fig. 4b), and no signal was found in uninfected water buffalo RBCs and pre-immune sera (data not shown). For further characterization of BoTRAP1, we performed an IFAT separately using anti-BoTRAP1 rabbit immune serum and pre-immune sera. The results showed that BoTRAP1 is localized on the apical end of intracellular or extracellular parasite under a confocal laser scanning microscope (Fig. 5a). No signals were observed in parasites when tested using pre-immune sera (Fig. 5b).

### Homology modeling of BoTRAP1

**Fig. 6:**
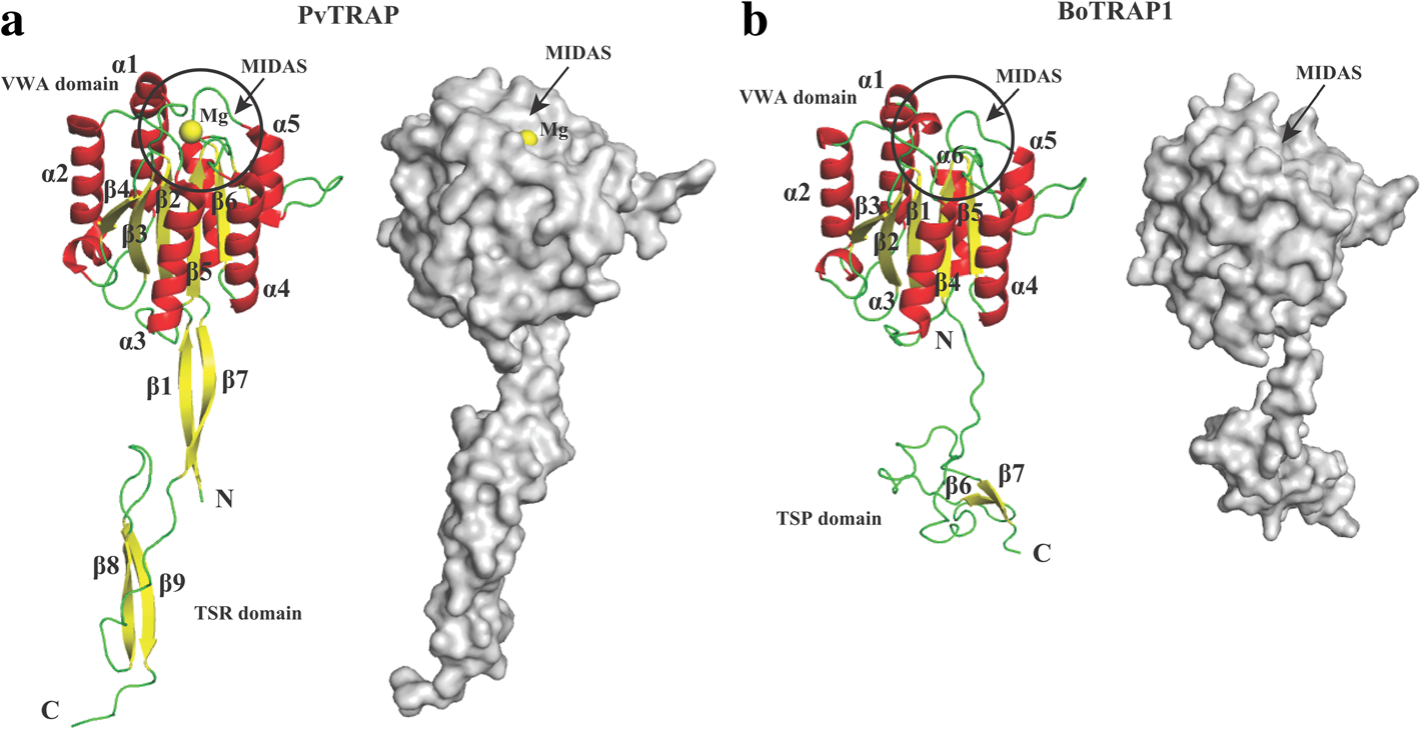
3D structure model of BoTRAP1 constructed by using SWISS-MODEL. **a** Crystal structure of PvTRAP (residues 25-283). The structure of PvTRAP is shown as a cartoon (left) and surface structure (right). The Mg^2+^ ion at the ligand binding site was colored in yellow. The α-helices were shown as red, and yellow indicates β-strands. **b** Predicted BoTRAP1 structure (residues 44-297). The 3D structure in identical orientations contains six α-helices (α1 and α6) and seven β-strands (β1 to β7). The BoTRAP1 vWFA domain also includes a metalion-dependent adhesion site (MIDAS) for binding ligand. The figure on the left shows the cartoon structure of BoTRAP1, and the figure on the right shows BoTRAP1 surface structure

To further confirm the potential role of BoTRAP1 in the invasion of parasites into host cells, the 3D structures of BoTRAP1 were homology-modeled using the crystal structure of *P*. *vivax* TRAP protein as a template. As shown in Fig. 6b, the predicted BoTRAP1 3D model contain vWFA and TSP-1 domains, with an inserted domain in the integrin of the vWFA domain, containing a metalion-dependent adhesion site (MIDAS) for interaction with ligand. While the amino acid sequences are not well conserved between BoTRAP1 and PvTRAP (28% similarity), their overall structures share a conserved feature (Fig. 6a, b).

## Discussion

Four TRAP genes have been identified in *B. bovis* and named *BbTRAP1-4* (GenBank accession numbers XM_001609738, XM_001609762, XM_001609736 and XM_001609760, respectively). The BbTRAPs share a typical structure with other apicomplexan TRAPs, including vWFA and TSP domains as well as a transmembrane region followed by a cytoplasmic C-terminal tail domain, except for the absence of the TSP domain from BbTRAP4 [19]. The TRAP family has also been identified in *B. orientalis* and named as TRAP1-4 (Data for TRAP2-4 not shown). Sequence analysis indicated that the four TRAP genes are very similar in both *B. bovis* and *B. orientalis*.

The TRAPs are secreted from micronemes as a transmembrane protein and are present in all apicomplexan parasites, with a high similarity for their cellular localization in *Plasmodium* spp., *Neospora caninum*, *B. bovis* and *B. gibsoni* [7, 10, 19, 20]. Confocal laser microscopy revealed that the native BoTRAP1 is located in the apical end of parasites, which is consistent with the TRAP localization of other parasites. On Western blot, the anti-rBoTRAP1 antibody detected the native protein as a ~65 kDa band from *B. orientalis* lysates. This protein size is in agreement with the theoretical mass of native BoTRAP1 (65 kDa) and is approximately the size of BbTRAP1 (73 kDa). Together, these results further verified that the sequence we obtained from *B. orientalis* cDNA precisely is the *BoTRAP1* gene.

Furthermore, we tried to determine the ectodomain crystal structure of BoTRAP1 (vWFA and TSP-1 domains). Despite a high purity and high concentration, the rBoTRAP1 failed to grow crystals in crystal buffer (576-well) (Hampton, California, USA). This result is probably attributed to the use of the prokaryotic, rather than the eukaryotic, expression system, leading to the absence of glycosylation on the surface of the recombinant protein. Fortunately, the overall structure of TRAP family is quite conservative across all apicomplexan parasites [21, 22]. For further characterization, the BoTRAP1 ectodomain structure was simulated by homologous modeling.

**Fig. 7:**
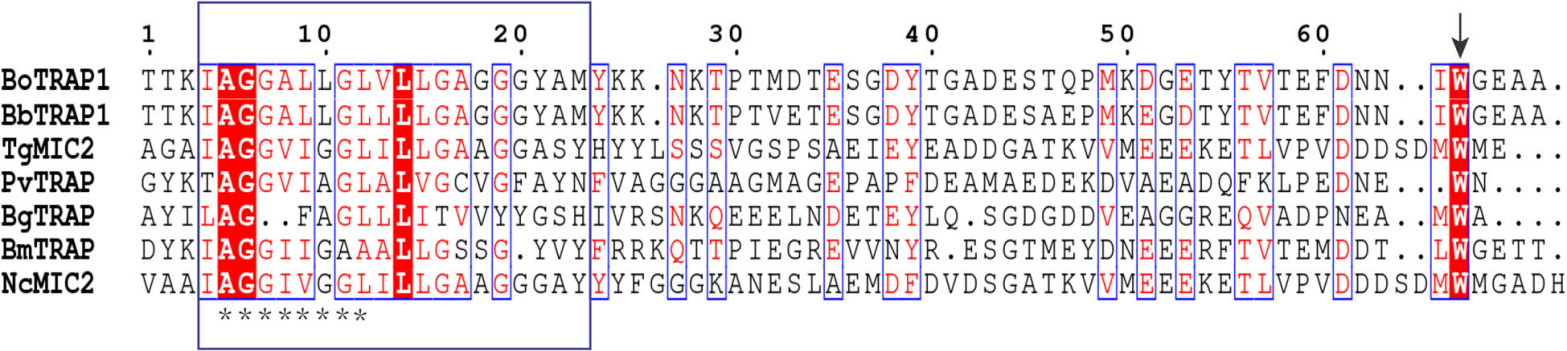
The predicted transmembrane regions and the cytoplasmic tails of apicomplexan TRAP-family proteins. The putative rhomboid cleavage sites are marked with asterisks, the predicted transmembrane domains by rectangles, and the sub-terminal tryptophan residues with an arrow

The interaction between the parasite ligands and the corresponding host-cell receptors is essential to the invasion of apicomplexan parasites [4, 7]. Currently, the invasion mechanism of apicomplexan parasites is poorly understood. A related study has shown that TRAPs participate in the formation of actin-myosin motor and drive the parasites into the host cells, enabling the translocation of TRAPs to the posterior pole of the parasites along the cytomembrane surface [23]. However, the actin-myosin motor was mediated by a sub-terminal tryptophan residue within the cytoplasmic tail of TRAPs, and the tryptophan residue connected to aldolase is rather conserved across apicomplexan parasites [4]. Interestingly, in order to release the parasites into host cells completely, the TRAPs translocated to the posterior end from the apical end of parasites need to be cleaved at their transmembrane domains by the rhomboid proteases (ROMs) [24, 25]. In this study, we predicted the transmembrane region and the putative rhomboid cleavage site of BoTRAP1, as well as detected the conserved tryptophan residue present, in the cytoplasmic tail domain (Fig. 7).

In malarial parasites, the GPI-linked protein semaphorin-7A (CD108) had been identified as an erythrocyte surface receptor for the *P. falciparum* MTRAP [26]. However, the erythrocyte receptor is still unknown for the BoTRAPs. Therefore, exploring the interaction of BoTRAPs with erythrocyte surface would facilitate the understanding of the invasion mechanism of *B. orientalis* and the discovery of new blocking agents or methods for controlling *B. orientalis*.

## Conclusions

Overall, BoTRAP1 was cloned, sequenced and expressed, with the native BoTRAP1 being characterized as ~65 kDa by Western blot and located in the apical end of the parasites. The simulation structure and functional analysis revealed that BoTRAP1 might perform critical functions in aiding parasite invasion and interaction of parasite-host RBCs. Blocking TRAP1 function might prevent the parasites from invading RBCs. Therefore, the newly identified and characterized BoTRAP1 may have potential as drug target or vaccine candidate antigen for preventing and controlling *B. orientalis* infection in water buffalo.

## Abbreviations

TRAP: thrombospondin-related anonymous protein
vWFA: Von Willebrand factor A
TSR: Thrombospondin type-1 repeat
CTD: Cytoplasmic tail domain
MIDAS: Metalion-dependent adhesion site
TEV: Tobacco Etch Virus
GPI: Glycosyl phosphatidylinositol anchor
PPE: Percentage of parasitized erythrocytes
PBS: Phosphate-buffered saline
DMSO: Dimethylsulfoxide
ROMs: Rhomboid proteases
RBC: Red blood cell
BSA: Bull serum albumin

## References

[1] Yao B, Zhao J, Liu E, Ding S, Shi J, Liu Z (2002). Serological investigations on *Babesia orientalis* infection. Status of water buffaloes in Hubei Province. Parasitol Res..

[2] Liu Z, Zhao J, Ma L, Yao B (1997). Studies on buffalo babesiosis in Hubei Province, China. Trop Anim Health Prod..

[3] Liu Q, Zhao J, Zhou Y, Liu E, Yao B, Fu Y (2005). Study on some molecular characterization of *Babesia orientalis*. Vet Parasitol..

[4] Morahan BJ, Wang L, Coppel RL (2009). No TRAP, no invasion. Trends Parasitol..

[5] Robson KJ, Dolo A, Hackford IR, Doumbo O, Richards MB, Keita MM (1998). Natural polymorphism in the thrombospondin-related adhesive protein of *Plasmodium falciparum*. Am J Trop Med Hyg..

[6] Templeton TJ, Kaslow DC (1997). Cloning and cross-species comparison of the thrombospondin-related anonymous protein (TRAP) gene from *Plasmodium knowlesi*, *Plasmodium vivax* and *Plasmodium gallinaceum*. Mol Biochem Parasitol.

[7] Baum J, Richard D, Healer J, Rug M, Krnajski Z, Gilberger TW (2006). A conserved molecular motor drives cell invasion and gliding motility across malaria life cycle stages and other apicomplexan parasites. J Biol Chem..

[8] Baum J, Gilberger TW, Frischknecht F, Meissner M. Host-cell invasion by malaria parasites: insights from *Plasmodium* and *Toxoplasma*. Trends Parasitol. 2008;24:557–63. 10.1016/j.pt.2008.08.006 18835222

[9] Gaffar FR, Yatsuda AP, Franssen FF, de Vries E (2004). A *Babesia bovis* merozoite protein with a domain architecture highly similar to the thrombospondin-related anonymous protein (TRAP) present in *Plasmodium* sporozoites. Mol Biochem Parasitol..

[10] Zhou J, Fukumoto S, Jia H, Yokoyama N, Zhang G, Fujisaki K (2006). Characterization of the *Babesia gibsoni* P18 as a homologue of thrombospondin related adhesive protein. Mol Biochem Parasitol..

[11] Sibley LD (2004). Intracellular parasite invasion strategies. Science..

[12] Soldati-Favre D (2008). Molecular dissection of host cell invasion by the apicomplexans: the glideosome. Parasite..

[13] Jones ML, Kitson EL, Rayner JC (2006). *Plasmodium falciparum* erythrocyte invasion: a conserved myosin associated complex. Mol Biochem Parasitol..

[14] Buscaglia CA, Coppens I, Hol WG, Nussenzweig V (2003). Sites of interaction between aldolase and thrombospondin-related anonymous protein in *Plasmodium*. Mol Biol Cell..

[15] Fish L, Leibovich B, Krigel Y, McElwain T, Shkap V (2008). Vaccination of cattle against *B. bovis* infection with live attenuated parasites and non-viable immunogens. Vaccine..

[16] Morgan RE, Evans KM, Patterson S, Catti F, Ward GE, Westwood NJ (2007). Targeting invasion and egress: from tools to drugs?. Curr Drug Targets..

[17] Nemetski SM, Cardozo TJ, Bosch G, Weltzer R, O’Malley K, Ejigiri I, et al. Inhibition by stabilization: targeting the *Plasmodium falciparum* aldolase-TRAP complex. Malar J. 2015;14:324. 10.1186/s12936-015-0834-9 PMC4545932 26289816

[18] He L, Feng H, Zhang Q, Zhang W, Khan MK, Hu M, et al. Development and evaluation of real-time PCR assay for the detection of *Babesia orientalis* in water buffalo (*Bubalus bubalis* Linnaeus, 1758). J Parasitol. 2011;97:1166–9. 10.1645/GE-2819.1 21711103

[19] Terkawi MA, Ratthanophart J, Salama A, AbouLaila M, Asada M, Ueno A (2013). Molecular characterization of a new *Babesia bovis* thrombospondin-related anonymous protein (BbTRAP2). PLoS One..

[20] Lovett JL, Howe DK, Sibley LD (2000). Molecular characterization of a thrombospondin-related anonymous protein homologue in *Neospora caninum*. Mol Biochem Parasitol..

[21] Song G, Koksal AC, Lu C, Springer TA (2012). Shape change in the receptor for gliding motility in *Plasmodium* sporozoites. Proc Natl Acad Sci USA..

[22] Song G, Springer TA (2014). Structures of the *Toxoplasma gliding* motility adhesin. Proc Natl Acad Sci USA..

[23] Menard R (2000). The journey of the malaria sporozoite through its hosts: two parasite proteins lead the way. Microbes Infect..

[24] Baker RP, Wijetilaka R, Urban S (2006). Two *Plasmodium* rhomboid proteases preferentially cleave different adhesins implicated in all invasive stages of malaria. PLoS Pathog..

[25] Brossier F, Jewett TJ, Sibley LD, Urban S (2005). A spatially localized rhomboid protease cleaves cell surface adhesins essential for invasion by *Toxoplasma*. Proc Natl Acad Sci USA..

[26] Bartholdson SJ, Bustamante LY, Crosnier C, Johnson S, Lea S, Rayner JC (2012). Semaphorin-7A is an erythrocyte receptor for *P. falciparum* merozoite-specific TRAP homolog, MTRAP. PLoS Pathog..

